# Operative hysteroscopy vs vacuum aspiration for treatment of retained products of conception: A systematic review and meta-analysis

**DOI:** 10.12669/pjms.42.8.16990

**Published:** 2026-08

**Authors:** Ping Qiu, Sisi Huang, Li Lu, Yingying Chen, Qing Wu

**Affiliations:** 1 Ping Qiu Department of Operating Room, Huzhou Maternity & Child Health Care Hospital, Huzhou, Zhejiang Province 313000, P.R. China; 2 Sisi Huang Department of Operating Room, Huzhou Maternity & Child Health Care Hospital, Huzhou, Zhejiang Province 313000, P.R. China; 3 Li Lu Department of Nursing, Huzhou Maternity & Child Health Care Hospital, Huzhou, Zhejiang Province 313000, P.R. China; 4 Yingying Chen Department of Science and Education, Huzhou Maternity & Child Health Care Hospital, Huzhou, Zhejiang Province 313000, P.R. China; 5 Qing Wu Department of Operating Room, Huzhou Maternity & Child Health Care Hospital, Huzhou, Zhejiang Province 313000, P.R. China

**Keywords:** Hysteroscopic surgery, Incomplete abortion, Residual trophoblastic tissue, Suction aspiration

## Abstract

**Objective:**

To generate comparative evidence on outcomes of hysteroscopic (HS) vs vacuum aspiration (VA) for retained products of conception (RPOC).

**Methodology:**

We included all study designs comparing HS with VA for RPOC and available on PubMed, CENTRAL, Scopus, Embase, and Web of Science up to 15^th^ January 2025. Surgical and pregnancy outcomes were analyzed.

**Results:**

Four studies were included. Meta-analysis showed that procedure duration was significantly longer with HS as compared to VA. The risk of intra-operative and post-operative complications did not differ between the two groups. The risk of incomplete evacuation of RPOC was significantly lower with HS as compared to VA with no difference in the risk of uterine perforation or hemorrhage ≥500ml. There was a tendency of higher risk of conversion to another procedure with HS as compared to VA. Meta-analysis showed that conception rates and subsequent risk of miscarriage did not significantly differ between the two groups.

**Conclusions:**

Limited evidence suggests that HS for RPOC may result in a lower risk of incomplete evacuation but has a longer procedural duration as compared to VA. The risk of complications and pregnancy outcomes do not seem to differ between the two. However, important fertility-related outcomes, including intrauterine adhesions, live birth rates, time to conception, and placental complications, could not be adequately assessed due to limited data. The quality of evidence is low due to scarce literature.

## INTRODUCTION

Retained products of conception (RPOC) or residual trophoblastic tissue can be found in the uterine cavity after term delivery, miscarriage, or termination of pregnancy in about 3% of all cases.^1^ Nevertheless, its prevalence can vary with some reports suggesting the occurrence of RPOC in 6% cases after pre-term birth or termination of pregnancy in the 1^st^ or 2^nd^ trimester and as high as 32% after medical abortions.^2^,^3^ RPOC can lead to several short-term complications like infections, and abnormal vaginal bleeding as well as long-term complications like intrauterine adhesions (IUAs), endometritis, Asherman’s syndrome and potentially causing subfertility.^4^ Patients often present with abnormal vaginal discharge or bleeding, and amenorrhea with or without pelvic pain.^5^ However, a significant portion of females may also be asymptomatic, making diagnosis challenging. Ultrasonography (USG) or color Doppler assessments are invaluable in arriving at a diagnosis of RPOC. USG evidence of intrauterine hyperechogenic focal mass, ill-defined endometrium-myometrium interface with or without increased endometrium endometrial thickness of ≥15 mm two weeks post-abortion or pregnancy loss is usually diagnostic of RPOC.^6^,^7^

There remain three primary modalities of treating RPOC namely, expectant management, medical therapy (anti-progestins/prostaglandins), or surgery.^8^ While early medical or expectant management can avoid surgery in 60% of females with RPOC, surgery is often needed in a significant number of patients to prevent long-term complications.^9^ Surgical procedures can either be dilation and curettage (D & C), vacuum aspiration (VA), or hysteroscopy (HS). Earlier, D & C was the only available surgical modality that was associated with a high risk of hemorrhage, uterine perforation, IUAs, and abnormal embryonic implantation.^4^ VA is slightly less traumatic than curettage and can be performed either under manual or USG guidance and is now considered the reference standard.

Nevertheless, the lack of direct visualization with VA is still a drawback that can cause uterine scarring and associated complications.^10^ HS is a minimally invasive approach that permits the management of intra-uterine pathologies via the insertion of a hysteroscope through the cervical canal into the endometrial cavity. Thus, in theory, HS for treating RPOC may lead to a reduction in complications and improve future conception rates.^11^ Several, single-arm studies have reported good results with HS for treating RPOC.^2^,^5^,^11^ However, no meta-analysis has compared outcomes of HS with VA for RPOC. We report the results of the first meta-analysis assessing surgical and pregnancy outcomes after treatment of RPOC with HS or VA.

## METHODOLOGY

This systematic review and meta-analysis were designed to include all study types conducted on women with RPOC either after miscarriage, termination of pregnancy, or after normal delivery. For inclusion, studies had to compare HS with VA and report operative or pregnancy outcomes. Operative outcomes included procedure duration, intra-operative or post-operative complications, incomplete evacuation, uterine perforation, hemorrhage ≥500ml, and conversion to another procedure. Pregnancy outcomes included conception rates and miscarriage rates. We did not include studies that did not compare the two procedures, did not report the above-mentioned outcomes, were only published as abstracts, or were editorials and correspondences.

Databases explored were PubMed, CENTRAL, Scopus, Embase, and Web of Science up to 15^th^ January 2025. Two reviewers independently performed the search with no restriction on the language of publications. Search strategy is shown in Supplementary Table-I. To eliminate the possibility of missing studies, Google Scholar and a bibliography of included studies were also explored.

**Supplementary Table-I:** Search strategy

***PubMed***
((“Hysteroscopy”[Mesh] OR hysteroscop*[tiab] OR “operative hysteroscopy”[tiab] OR “hysteroscopic surgery”[tiab] OR “uterine endoscopy”[tiab] OR uteroscopy[tiab]) AND (“Retained Products of Conception”[tiab] OR RPOC[tiab] OR “retained product* of conception”[tiab] OR “retained trophoblastic tissue”[tiab] OR “residual trophoblastic tissue”[tiab] OR “retained placental tissue”[tiab] OR “Incomplete Abortion”[Mesh] OR “incomplete abortion”[tiab] OR “incomplete miscarriage”[tiab]) AND (“Vacuum Curettage”[Mesh] OR “vacuum aspiration”[tiab] OR “suction aspiration”[tiab] OR “manual vacuum aspiration”[tiab] OR “electric vacuum aspiration”[tiab] OR “ultrasound-guided vacuum aspiration”[tiab]))
***Embase***
(‘hysteroscopy’/exp OR hysteroscop*:ti,ab OR ‘operative hysteroscopy’:ti,ab OR ‘hysteroscopic surgery’:ti,ab OR ‘uterine endoscopy’:ti,ab OR uteroscopy:ti,ab) AND (‘retained products of conception’:ti,ab OR rpoc:ti,ab OR ‘retained trophoblastic tissue’:ti,ab OR ‘residual trophoblastic tissue’:ti,ab OR ‘retained placental tissue’:ti,ab OR ‘incomplete abortion’/exp OR ‘incomplete abortion’:ti,ab OR ‘incomplete miscarriage’:ti,ab) AND (‘vacuum aspiration’/exp OR ‘vacuum curettage’/exp OR ‘vacuum aspiration’:ti,ab OR ‘suction aspiration’:ti,ab OR ‘manual vacuum aspiration’:ti,ab OR ‘electric vacuum aspiration’:ti,ab)
***CENTRAL***
(hysteroscop* OR “operative hysteroscopy” OR “hysteroscopic surgery” OR “uterine endoscopy” OR uteroscopy) AND (“retained products of conception” OR RPOC OR “retained trophoblastic tissue” OR “residual trophoblastic tissue” OR “retained placental tissue” OR “incomplete abortion” OR “incomplete miscarriage”) AND (“vacuum aspiration” OR “suction aspiration” OR “manual vacuum aspiration” OR “electric vacuum aspiration”)
***Scopus***
TITLE-ABS-KEY(hysteroscop* OR “operative hysteroscopy” OR “hysteroscopic surgery” OR “uterine endoscopy” OR uteroscopy) AND TITLE-ABS-KEY(“retained products of conception” OR RPOC OR “retained trophoblastic tissue” OR “residual trophoblastic tissue” OR “retained placental tissue” OR “incomplete abortion” OR “incomplete miscarriage”) AND TITLE-ABS-KEY(“vacuum aspiration” OR “suction aspiration” OR “manual vacuum aspiration” OR “electric vacuum aspiration”)
***Web of Science***
TS=(hysteroscop* OR “operative hysteroscopy” OR “hysteroscopic surgery” OR “uterine endoscopy” OR uteroscopy) AND TS=(“retained products of conception” OR RPOC OR “retained trophoblastic tissue” OR “residual trophoblastic tissue” OR “retained placental tissue” OR “incomplete abortion” OR “incomplete miscarriage”) AND TS=(“vacuum aspiration” OR “suction aspiration” OR “manual vacuum aspiration” OR “electric vacuum aspiration”)

We combined the search results, deduplicated them, and then examined each study for further full-text analysis based on information in the title and abstract. Complete texts of selected studies were checked before final inclusion. Disagreements were resolved by mutual discussion till a consensus was reached.

We reported the review based on Preferred Reporting Items for Systematic Reviews and Meta-Analyses (PRISMA) guidelines for this study.^12^ The study protocol was registered on the PROSPERO database (CRD42025636621).

### Data management:

The same two reviewers extracted information from the studies which included: first author, location, year, sample size, included population, type of HS intervention, age, gravida, parity, body mass index, asymptomatic cases, RPOC size, follow-up, and outcomes. The data was extracted independently by the two reviewers and re-checked for errors. Outcomes examined in the review included: procedure duration, intra-operative or post-operative complications, incomplete evacuation, uterine perforation, hemorrhage ≥500ml, conversion to another procedure, conception rates, and miscarriage rates.

### Quality assessment:

Randomized controlled trials (RCTs) were examined using the Cochrane Collaboration risk of bias-2 tool.^13^ Studies were judged for the randomization process, deviation from intended intervention, missing outcome data, measurement of outcomes, selection of reported results, and overall risk of bias. The quality of cohort studies was examined based on the Newcastle Ottawa Scale (NOS).^14^ Stars were awarded for the selection of the study cohort, comparability of groups, and outcome assessment.

### Statistical analysis:

Analysis was done on “Review Manager” (RevMan, version 5.3). The duration of the procedure was a continuous outcome which was combined to generate Mean Difference (MD) and 95% confidence intervals (CI). Median (interquartile range) was converted to mean (standard deviation) by the methods of Wan et al.^15^ All other outcomes were dichotomous and pooled as odds ratio (OR). Publication bias was not examined due to small number of studies. Due to very small number of studies, sensitivity analyses was not performed. The chi-square-based Q statistics and I^2^ statistics were used for inter-study heterogeneity. A p-value of <0.10 for Q statistic and I^2^ >50% meant substantial heterogeneity.

## RESULTS

**Fig.1:**
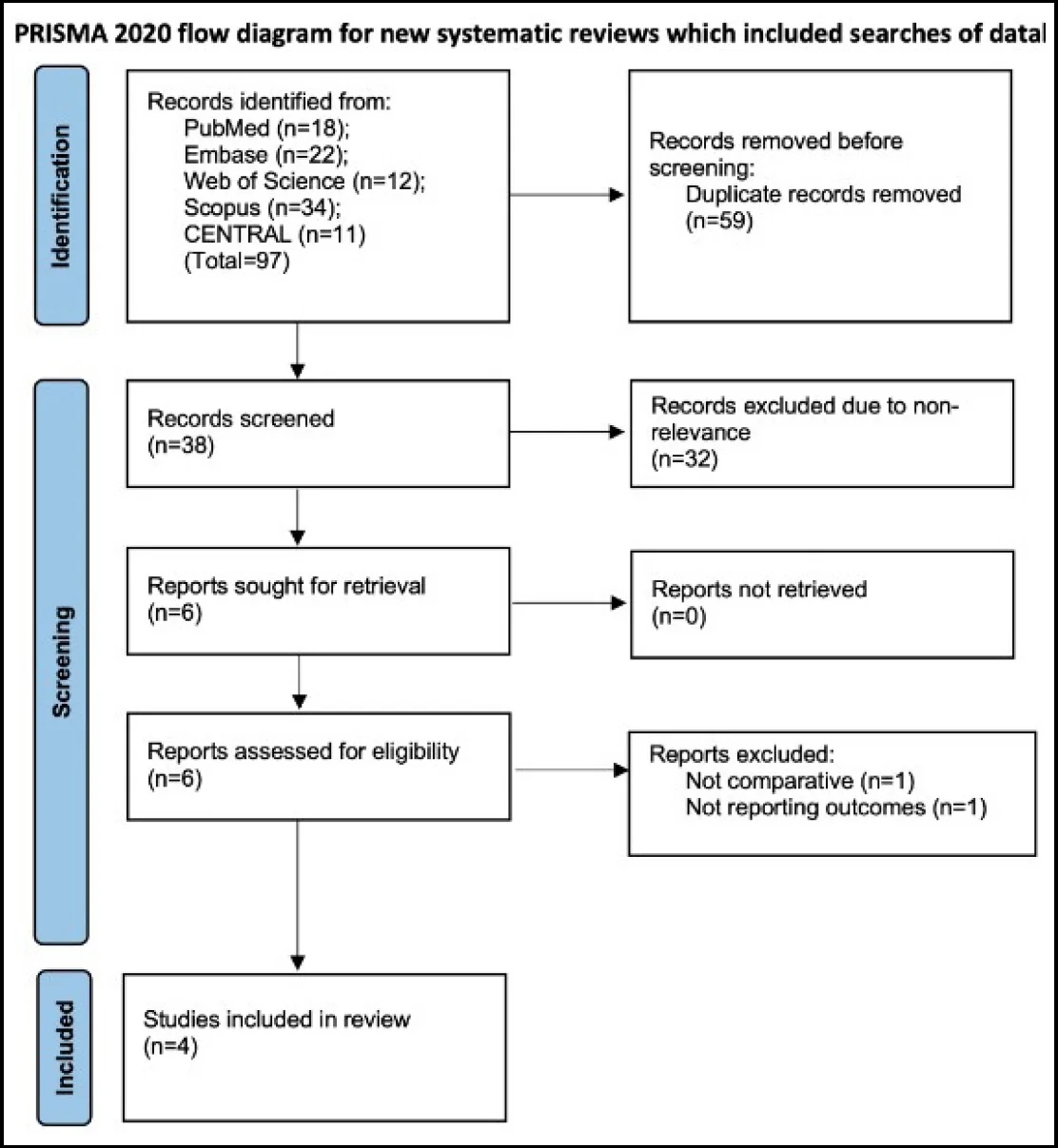
Flowchart of the study.

The literature search on all databases led to a total of 97 articles (Fig.1). Fifty-nine duplicate studies were removed. The remaining 38 articles were assessed for eligibility and of these six were selected for complete text review. Four studies^16^–^19^ was included in the meta-analysis.

The baseline details of the studies can be found in Table-I. There were two RCTs, one prospective and one retrospective cohort study. Wagenaar et al. conducted an RCT^19^ and observational study^18^ parallelly in the same healthcare setups. They also reported pregnancy outcomes of both studies in a separate article,^20^ data of which was also included in the meta-analysis. Wagenaar et al.^18^,^19^ used USG guidance for VA in both their studies while only manual VA was performed in the study of Karavani et al.^16^ The reviewers ensured that patients were not counted twice in these publications. In the RCT of Huchon et al.,^17^ the choice of USG was at the discretion of the treating clinician. The HS technique was morcellation only in the RCT of Wagenaar et al.^19^, both cold loop and morcellation in the cohort study^18^ of the same authors, and cold loop only in the remaining studies.^16^,^17^ The smallest study was that of Karavani et al.^16^ while the largest was that of Huchon et al.^17^ About 21 to 42 percent of females were asymptomatic in the included studies. The quality assessment of studies is shown in Table-II. Both RCTs were considered to have a high risk of bias due to concerns regarding deviation from the intended intervention, lost to follow-up, and lack of blinding. Both cohort studies also scored low on the NOS receiving only six stars each.

**Table-I:** Information retrieved from the included studies.

Study & location	Design	Eligible participants	HS intervention	Gro-ups	Sample size	Age (ye-ars)	BMI (kg/m^2^)	Previous CS (%)	Gravidity	Parity	Asym-ptomatic (%)	RPOC size (mm)	Follow-up
Wagenaar 2023; Netherlands & Belgium	PC	USG suggestive of RPOC ranging from 1 to 4 cm after miscarriage, termination of pregnancy, or delivery	Morcellation or cold loop	HS VA	124 28	32 32	21 [21-26.4] 24 [22.4-26]	10.5 3.6	2 [1-3] 2 [1-3]	1 [0-2] 1 [0-2]	31.5 21.4	20± 8 27± 8	55 months
Wagenaar 2023; Netherlands & Belgium	RCT	USG suggestive of RPOC ranging from 1 to 4 cm after miscarriage, termination of pregnancy, or delivery	Morcellation	HS VA	87 86	32 32	23 [21 – 25] 24 [21 – 26]	11.5 12.8	2 [1-3] 2 [1-3]	1 [0-2] 1 [0-2]	21.8 23.5	20 [15-25] 20 [15-28]	55 months
Huchon 2023; France	RCT	USG suggestive of RPOC <5cm after incomplete spontaneous abortion	Cold loop	HS VA	282 281	33.2 32.1	NR	15 18	2 [2-4] 2 [2-4]	1 [0-2] 1 [0-2]	37 42	NR	2 years
Karavani 2017; Israel	RC	RPOC after normal vaginal delivery	Cold loop	HS VA	63 23	31.7 30	NR	NR	NR	NR	22.2 21.7	22.2± 10.5 23.8± 9.4	30.9- 48.6 months

RCT, randomized controlled trial ; RC, retrospective cohort; USG, ultrasonography; RPOC, retained products of conception; HS, Hysteroscopy; VA, vacuum aspiration; PC, prospective cohort; wks, weeks; NOS, Newcastle Ottawa scale.

**Table-II:** Risk of bias analysis.

Clinical trials^#^	Randomization process	Deviation from intended intervention	Missing outcome data	Measurement of outcomes	Selection of reported result	Overall risk of bias
Huchon 2023	Low risk	Some concerns	Some concerns	Low risk	Low risk	High risk
Wagenaar 2023	Low risk	Low risk	Some concerns	Some concerns	Low risk	High risk
Observational studies^	Selection of cohort			Comparability of groups		Outcome assessment
Wagenaar 2023	****			-		**
Karavani 2017	****			-		**

# Based on the Cochrane risk of bias-2 tool ^Based on the Newcastle Ottawa scale.

Stars were awarded for each domain of the scale.

### Outcomes

**Fig.2:**
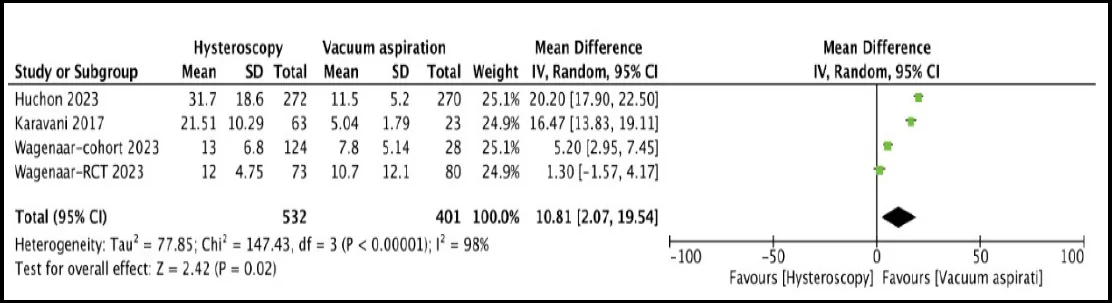
Meta-analysis of procedural duration between HS and VA groups.

**Fig.3:**
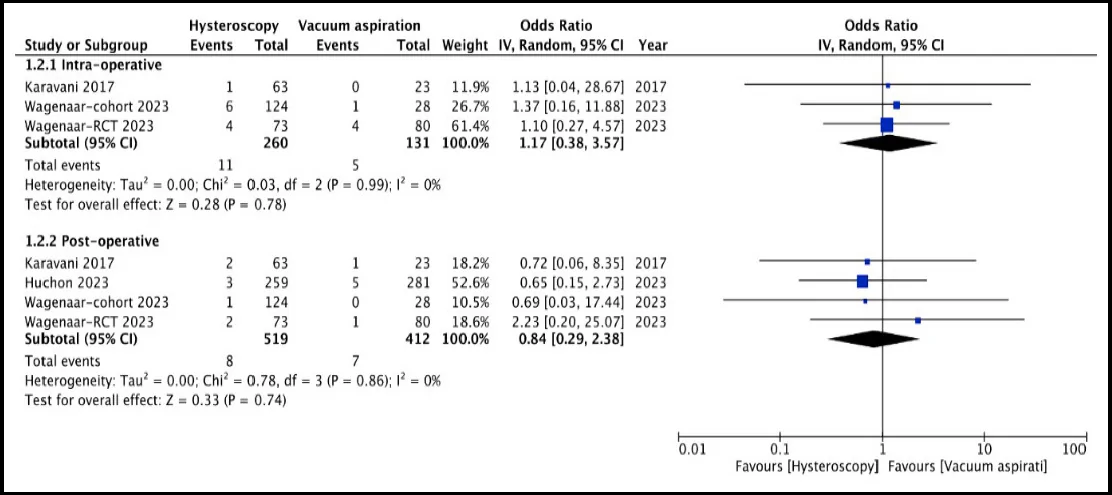
Meta-analysis of complication rates between HS and VA groups.

**Supplementary Fig.1:**
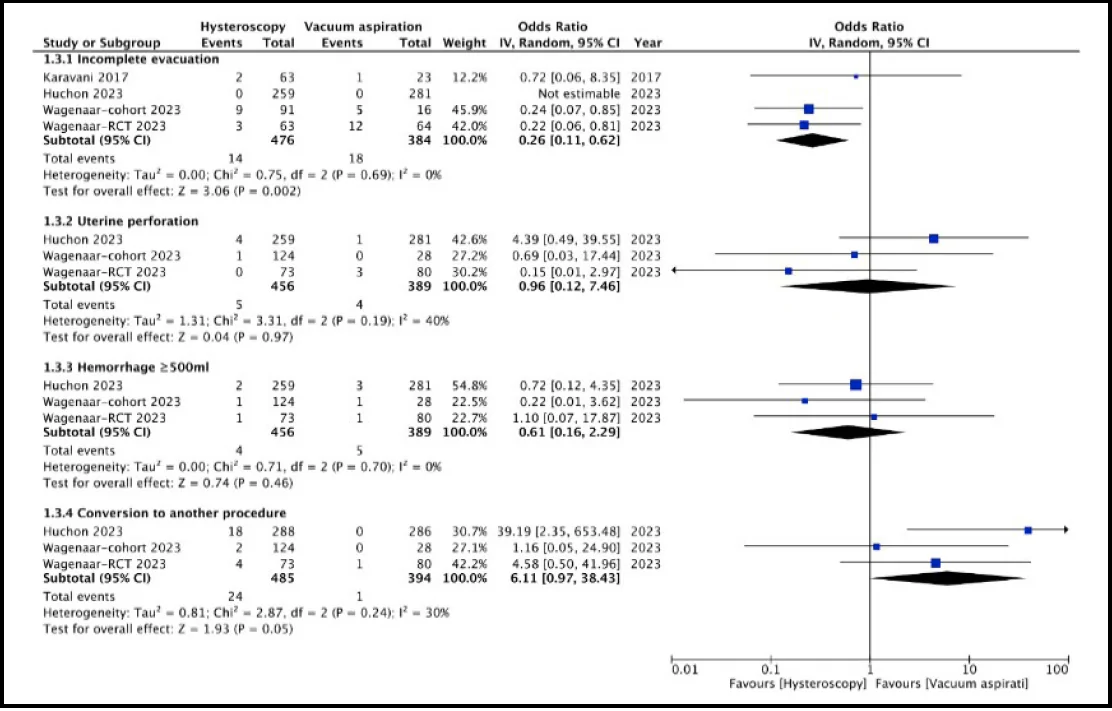
Meta-analysis of incomplete evacuation, uterine perforation, hemorrhage ≥500ml, and conversion to another procedure between HS and VA groups.

Meta-analysis showed that procedure duration was significantly longer with HS as compared to VA (MD: 10.81 95% CI: 2.07, 19.54 I^2^=98%) (Fig.2). However, the risk of intra-operative (OR: 1.17 95% CI: 0.38, 3.57 I^2^=0%) and postoperative complications (OR: 0.84 95% CI: 0.29, 2.38 I^2^=0%) did not differ between the two groups (Fig.3). The risk of incomplete evacuation of RPOC was significantly lower with HS as compared to VA (OR: 0.26 95% CI: 0.11, 0.62 I^2^=0%) (Supplementary Fig.1). However, there was no difference in the risk of uterine perforation (OR: 0.96 95% CI: 0.12, 7.46 I^2^=40%) or hemorrhage ≥500ml (OR: 0.61 95% CI: 0.16, 2.29 I^2^=0%) between the two groups (Supplementary Fig.1). There was a tendency of higher risk of conversion to another procedure with HS as compared to VA (OR: 6.11 95% CI: 0.97, 38.43 I^2^=0%) (Supplementary Fig.1).

**Supplementary Fig.2:**
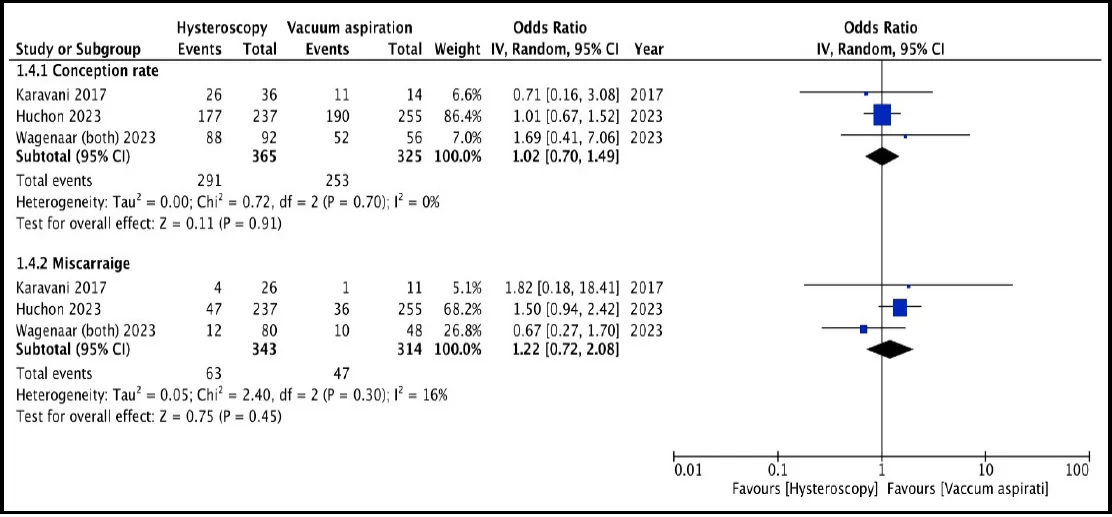
Meta-analysis of conception rates and miscarriage between HS and VA groups.

Pregnancy outcomes are presented in Supplementary Fig.2. A large number of females were lost to follow-up or did not have a desire to conceive in the included studies, hence the final number of included females for pregnancy outcomes was lower. Meta-analysis showed that conception rates (OR: 1.02 95% CI: 0.70, 1.49 I^2^=0%) and subsequent risk of miscarriage (OR: 1.22 95% CI: 0.72, 2.08 I^2^=16%) did not significantly differ between the two groups.

## DISCUSSION

We have presented the results of the first systematic review and meta-analysis comparing surgical and pregnancy outcomes following management of RPOC with either HS or VA. Comparing data from a limited number of studies with varying study designs, it was noted that HS had significantly longer procedure duration as compared to VA. The overall MD was found to be 10.8 minutes favoring VA, however with high inter-study heterogeneity, thereby diminishing the reliability of outcomes. The difference could be due to additional steps involved in the installation of the HS system which can be reduced with simplification of steps and regular practice. In many clinical settings, an additional 10 minutes of operative time may not be clinically relevant. This small increase may be acceptable if it translates into advantages like direct visualization of the uterine cavity and a lower risk of incomplete evacuation. However, in very high-volume centers or resource-limited settings, a 10 minute increase may affect operating room efficiency, scheduling, costs, and anesthesia exposure. It is also important to recognize that procedural duration may be influenced by operator experience and the specific hysteroscopic technique employed. Therefore, while HS seems to require more operative time than VA, the clinical significance of this difference should be assessed in terms of resource availability. Secondly, we also found a significantly lower risk of incomplete evacuation with HS as compared to VA. This is in line with published literature.

In a systematic review, Hooker et al.^21^ have found that HS results in significantly higher rates of complete evacuation of RPOC as compared to D & C (99% vs 71%, respectively). It can be noted that both D & C and VA are relatively blind procedures, except for use of USG guidance in some cases with VA. The direct vision offered by HS allows the clinician to completely visualize if RPOC, especially adherent tissue, is completely evacuated. However, the time interval between diagnosis and intervention can be an important confounder. In the studies of Wagenaar et al.,^18^,^19^ time interval for HS was significantly longer as compared to VA. Longer duration can lead to reduction in the size of RPOC and make the tissues less adherent.

The rate of both intra-operative and post-operative complications were not found to be significantly different between HS and VA. Results may be interpreted with caution given the small number of studies available for review. Assessing specific complications, there was no difference in the risk of uterine perforation or hemorrhage ≥500ml between the two groups indicating that in terms of safety there may be no difference between either modalities. We noted a 4.2% risk of intra-operative complications, 1% risk of uterine perforation, and 0.8% risk of hemorrhage ≥500ml with HS in our review. In comparison, a systematic review^5^ of single-arm studies has shown a pooled incidence of 1.9% for intra-operative complications and 1.1% for uterine perforations with HS.

We also noted a tendency of increased conversion of HS to VS in our meta-analysis but results were not statistically significant. Conversion was usually performed in the studies due to any intra-operative adverse event or inability to remove the RPOC. It can be seen on the forest plot that the results were highly influence by the trial of Huchon et al.^17^ which reported a large number of conversions in the HS group due to reasons mentioned earlier of all complications, the risk of IUAs is the most significant. IUAs can cause menstrual disturbances, subfertility, and recurrent pregnancy loss along with obstetrical complications like placental abnormalities and preterm delivery.^22^ Due to limitation of data, a meta-analysis could not be performed for IUAs. However, IUAs were reported by two studies. The RCT of Wagenaar et al^19^ reported no difference in the risk of IUAs with HS or VA (14.3% vs 20.6%, respectively). In the cohort study of the same authors, similar rate of IUAs was found in the HS group (15.4%) but the risk was lower in the VA group (6.3%) which could be due to the small number of participants in the latter. In comparison, a meta-analysis of eight single-arm studies have showed that the risk of IUAs with HS is around 6.8% ranging from 2.6% to 12.4%.^5^ These rates are much lower than those noted after D & C at 29.6%.^22^ Another study by Chung et al.^23^ has compared USG guided VA and manual VA for treating delayed or incomplete miscarriage of ≤12 weeks of gestation. The authors showed that the risk of IUAs was significantly lower with USG guided VA (19%) compared to electric VA (32%). In the four included studies, both manual and USG guided VA were used and limited data was available on the risk of IUAs. More studies comparing the two modalities and reporting IUAs are needed for generating high-quality comparative evidence.

Subsequent pregnancy outcomes after treatment of RPOC are as important as surgical outcomes for the choice of the procedure. We were able to compare future conception and miscarriage rates in our meta-analysis, albeit with a lower sample size as only females desirous of pregnancy and on long term follow-up were included. The meta-analysis failed to demonstrate a statistically significant difference in the analyzed pregnancy outcomes. A more detailed analysis of pregnancy outcomes was conducted by Wagenaar et al.^20^ wherein they reported no difference in live birth rates, time to conception, or pregnancy complications in the first subsequent pregnancy after HS or VA. A meta-analysis^5^ of single arm studies has also shown high conception rate of 81.1% after HS for RPOC but low rates for those undergoing D & C (65.4%). We acknowledge that data is scarce at this point and only further studies can decipher differences in pregnancy outcomes between the two groups.

The present review had high heterogeneity in most of the results. Indeed, there were several sources of clinical heterogeneity between the studies. The included studies differed in the hysteroscopic techniques employed, with some using morcellation while others utilized cold loop resection. Furthermore, studies varied in terms of USG use for VA procedures. USG can improve the procedural accuracy and potentially lower rates of IUA compared with blind techniques and this may have introduced heterogeneity. The indications for surgery also varied, with some including RPOC following miscarriage, termination of pregnancy, and delivery. This may represent populations with different baseline risks. Additionally, there were differences in the interval between diagnosis and intervention could influence the size and adherence of retained tissue, potentially affecting procedural success and complication rates. Finally, follow-up durations also varied between studies ranging from two years to over 55 months, which may have impacted long-term reproductive outcomes. Due to the limited number of available studies, subgroup analyses to explore the influence of these factors were not possible. Consequently, these sources of heterogeneity reduce the certainty of the evidence.

### Limitations:

Other than the small number of studies, there are several other limitations which should be considered for this review. Varying study designs is another drawback as only two were RCTs while another two were cohort studies which had obvious selection bias. However, literature indicates that some of these variables may not affect outcomes. Melcer et al.^24^ have shown that there is no difference in reproductive outcomes of women with RPOC following spontaneous vaginal delivery or first-trimester pregnancy termination. An RCT comparing HS morcellation with loop resection has shown that the former is faster but both techniques are equally safe and have high rates of complete removal.^25^ While this may provide some certainty to the results, the numerous other confounders still downgrade the quality of the evidence. Moreover, the risk of bias in the included studies was high, both in cohort as well as RCTs. We were also unable to examine important outcomes like grades of complications, IUA, time to conception, etc. due to lack of sufficient data. Several of the included studies failed to report baseline characteristics which prevented comparison across groups. Lastly, a formal assessment of publication bias could not be assessed due to the small number of available studies. Furthermore, although a formal GRADE assessment was not conducted, the overall certainty of evidence for the evaluated outcomes appears to be low to very low. This judgment is based on several factors, like the limited number of studies, inclusion of observational designs alongside randomized trials, high risk of bias within the included studies, clinical heterogeneity, and imprecision in effect estimates. Therefore, the findings of this review should be interpreted cautiously.

Nevertheless, we believe that our study still presents important comparative evidence to allow clinicians to make informed decisions while treating women with RPOC. At this point the choice of either technique should be based on patients’ characteristics, costs, as well as patient preferences. Patients should be informed about the current preliminary evidence along with its limitations to allow informed decisions. The information obtained in our review is also important for obstetric nursing who play a critical role in supporting hysteroscopy procedures, ensuring patient safety, comfort, and the smooth operation of the procedure. Their responsibilities span before, during, and after the procedure, including both clinical and emotional aspects of care. Nursing personnel may use such information to provide appropriate counselling to women undergoing treatment for RPOC. Moreover, the results of our review may also assist planning of future studies. Robust RCTs taking into account distinct patient characteristics, HS and VA techniques, and long follow-up are needed to provide high-quality evidence for important subgroups.

## CONCLUSIONS

Limited evidence with high heterogeneity suggests that HS for RPOC may result in a lower risk of incomplete evacuation but has a longer procedural duration as compared to VA. The risk of complications and pregnancy outcomes do not seem to differ between the two. However, current evidence is insufficient to establish whether HS is better in preserving fertility. Important reproductive outcomes, including intrauterine adhesions, live birth rates, time to conception, and placental complications, remain inadequately studied. The quality of evidence is low due to scarce literature.

### Authors’ contributions:

**PQ:** Literature search, was contributed to the study design and manuscript writing.

**SH, LL, YC and QW:** data collection, data analysis and interpretation. Critical review.

**PQ:** Manuscript revision and validation and is responsible for the integrity of the study.

All authors have read and approved the final manuscript.
